# Rosiglitazone alleviates myocardial apoptosis in rats with acute myocardial infarction via inhibiting TLR4/NF-κB signaling pathway

**DOI:** 10.3892/etm.2021.10343

**Published:** 2021-06-29

**Authors:** Hongzhong Ma, Juan Du, Xiaoli Feng, Yingjun Zhang, Haihuan Wang, Suchun Ding, Aijie Huang, Jiahai Ma

Exp Ther Med 19:2491–2496, 2020; DOI: 10.3892/etm.2020.8479

Following the publication of this paper, it was drawn to the Editors' attention by a concerned reader that a comparison among the three separate panels in Fig. 3A showing apoptotic data revealed striking similarities in terms of the presentation of groupings of cells within the panels, such that it would be difficult to rationalize how the three sets of data could have been generated from discretely performed experiments. After having investigated the matter internally, the Editor of *Experimental and Therapeutic Medicine* has decided that this paper should be retracted from the Journal on account of a lack of confidence in these presented data. The authors proved not to be contactable to comment on these issues. The Editor apologizes to the readership for any inconvenience caused.

